# Translating medical device innovations to market - a Ugandan perspective

**DOI:** 10.1186/s13104-023-06541-6

**Published:** 2023-10-09

**Authors:** Brian Matovu, Jackline Winfred Baluka, Mercy Takuwa, Lucy Kevin Namuli, Charles Norman Mpaata, Julius Mugaga, Benedict Mulindwa, Racheal Nalwoga, Maria K Wolters, Robert Tamale Ssekitoleko

**Affiliations:** 1 Biomedical Engineering Unit, Department of Physiology, School of Biomedical Sciences, College of Health Sciences, Makerere University, Kampala, Uganda; 2 Institute of Design Informatics, School of Informatics, University of Edinburgh, Edinburgh, UK

**Keywords:** Medical devices, Medical device innovation, Medical device translation, Medical device regulation, Translational pathways, Interdisciplinary collaborations

## Abstract

There is a disparity between low and middle-income countries (LMICs) and high-income countries (HICs) in translating medical device innovations to the market, affecting health care service delivery. Whereas medical technologies developed in HICs face substantial challenges in getting to the bedside, there are at least clear pathways in most of the major markets, such as the UK, the EU, and the USA. Much less is known about the challenges that innovators of medical technologies face in LMICs. The aim of this study was to map out current bottlenecks in medical device innovation in Uganda, a LMIC in Sub-Saharan East Africa.

A cross-sectional survey was carried out using a digital questionnaire. Twenty-one individuals completed the questionnaire, with the majority being medical device innovators (n = 12). Only one of these had undertaken all the innovation stages, up to clinical validation. Very few innovators had established companies, and/or acquired intellectual property. It is evident from similar studies that challenges in medical device translations are multidimensional, and hence interdisciplinary collaborations are key to accelerating translation processes, especially for LMICs.

**Supplementary Information:**

The online version contains supplementary material available at 10.1186/s13104-023-06541-6.

## Introduction

There is a growing global demand and market for medical devices, which presents various opportunities for national economic growth and development [1]. However, these opportunities have been clustered in high-income countries (HICs) where the development of medical devices has been concentrated [2]. This explains why HICs with well-developed policies on innovation translation systems and processes continue to be the primary source of medical device innovations. Unfortunately, innovations from low- and middle income countries (LMICs) still face numerous challenges in their translation, which means that a far greater proportion of them remain on the shelf without reaching the market.

Some of the main challenges to innovation and translation in LMICs include high production costs, poor enabling infrastructure, and a lack of technological know-how among regulators and policymakers [3, 4]. Policies, guidelines, and regulations governing the translation of medical device innovations in LMICs may be unclear, not easy to find, or missing entirely [5]. Additionally, most LMICs lack R&D infrastructure and the funds needed to clinically validate medical devices, causing a gap in healthcare service innovation and preventing users from reaping the benefits of innovators’ creativity. Without addressing those gaps, the targets of Sustainable Development Goal 3 (Healthy Lives and Wellbeing for All Ages) cannot be met.

The aim of this study was to identify bottlenecks in medical device innovation translation in Uganda from the perspective of medical device innovators and stakeholders that support or regulate such innovation. It is part of a wider development of supporting infrastructure and favorable regulations. This would facilitate the adoption of locally developed medical devices that possess the key qualities of affordability, usability, and desirability [6].

## Main text

### Methods

We used a digital questionnaire (supplementary material 1) to obtain views on major issues in medical device translation. This questionnaire was developed by the principal investigator together with the research assistants under the Centre for Design Innovation and Translational excellence at Makerere University. This was later validated and piloted by a pool of experts from academia and industry. Participants were medical device innovators with a medical and/or engineering background as well as social scientists, business experts, and legal personnel with expressed interest for medical device innovation. Participants from outside the medical technology landscape were excluded from this study. The questionnaire was implemented in KoBoToolbox Non-Humanitarian Server Version 2.022.08. It was shared via email and through WhatsApp Messenger with a large network of stakeholders.

### Participants

Thirty-two individuals opened the questionnaire. Eleven individuals declined to consent. The remaining 21 participants completed to the questionnaire and were considered in the analysis. Of these, 52% (n = 11) were from academia and 48% (n = 10) from industry.

**Fig. 1:**
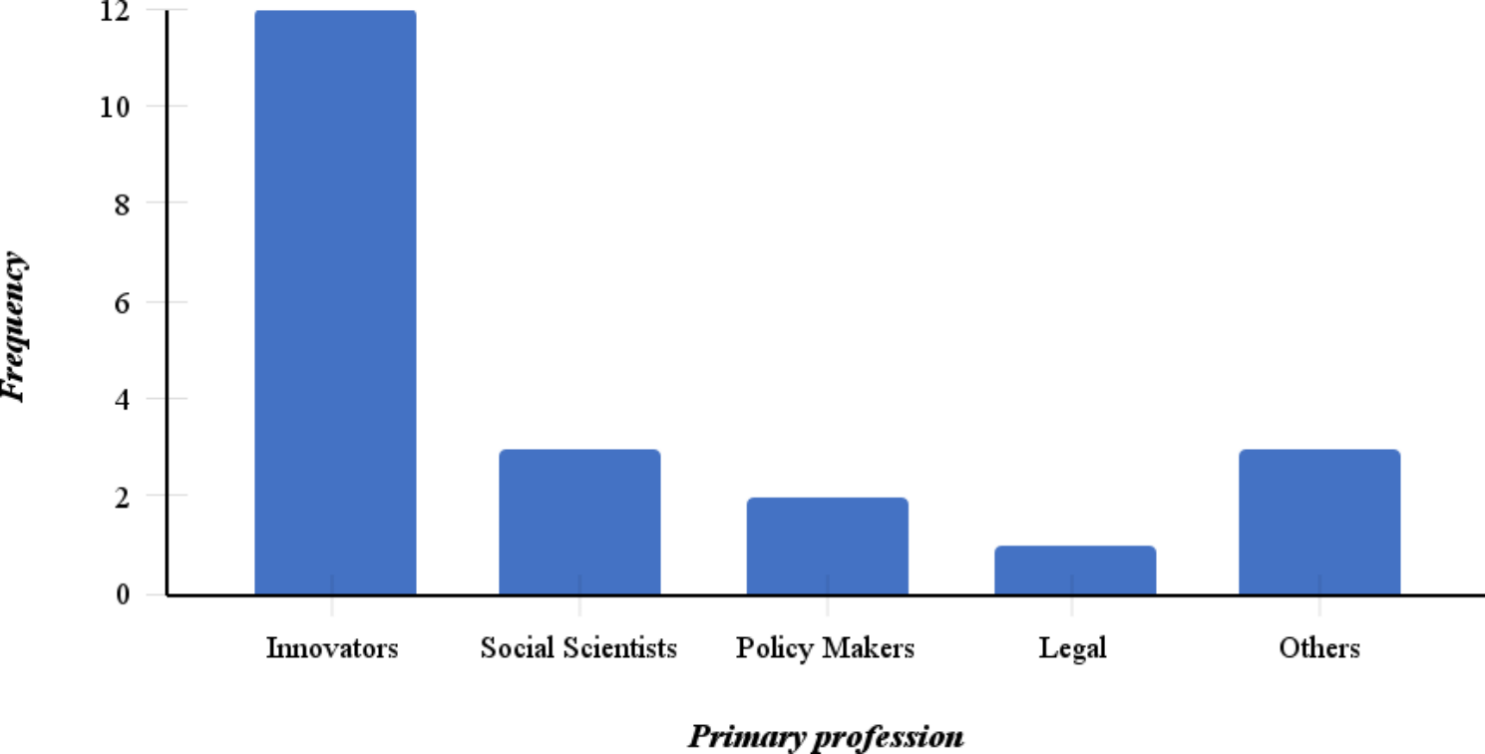
Primary profession categories of respondents

Participants were professionally categorized as innovators, social scientists, policymakers, or legal practitioners, and others, with 57% (n = 12) of the respondents identifying as innovators. Figure 1 below shows the primary professional categories of the 21 respondents.

Each of the 12 innovators was leading an innovation team and actively participating in the different stages of the innovation cycle. Six of the teams had only engineers, while the rest were multidisciplinary, having clinicians, academicians, and business specialists. For innovations to have a chance at moving to the market, engineers should seek to use a multidisciplinary approach in their teams [7]. That way, innovators can navigate some of the social, legal, and business issues such as safety, affordability, end user interaction, intellectual property (IP), and approvals that may act as hindrances to translation [8].

The majority of innovators had reached the proof-of-concept stage, an early innovation stage, with only one out of the respondents undertaking all the phases of development (ideation, conceptualization, and prototyping), preclinical stages, and clinical validation [9]. The results show that most innovators spent between 1 and 2 years at the proof-of-concept stage, while others spent over five years at the minimum viable product and clinical validation stages (Table 1).

**Table 1:** Stage of Innovation and number of years spent

Stage of innovation:	N (%)
Ideation and conceptualization	2 (17)
Proof of concept/Prototype	7 (58)
Preclinical validation	1 (8)
Minimum Viable Product	1 (8)
Clinical Validation	1 (8)
Premarket entry	0 (0)
Post market	0 (0)
**Total**	**12 (100)**
**No. of years**:	**N (%)**
Less than one year	3 (25)
1–2 years	5 (41)
2–3 years	2 (17)
3–4 years	0 (0)
4–5 years	0 (0)
Above 5 years	2 (17)
**Total**	**12 (100)**

### Regulations governing the translation of medical devices

Although respondents had a clear idea of how medical device regulation should be incorporated into the development process, they were uncertain of the existing support regulations and policies. This challenge is well-documented for Uganda and many other LMICs [10]. A study by Hubner et al. showed that 50% of the member countries of the College of Surgeons of East, Central, and Southern Africa did not have a formal process to regulate medical devices [11], even with the existence of international platforms by the WHO to facilitate the adoption from HICs. Matovu et al. described how unclear the clinical regulatory process for investigational medical devices is in Uganda and how this is affecting clinical trials, which is a critical stage of validation, and this is confirmed by our findings [12].

For example, Mpaata et al. in 2023 reported that one respondent provided a useful suggestion “*Review and expand the mandate of the National Drug Policy and Authority Act, Cap. 206, of 1993* [13], *to include medical devices and provide a basis for guidelines and regulations for innovation, development, use, and maintenance of medical devices.*” [22].

The *National Drug Policy and Authority Act, Cap. 206, of 1993*, establishes the National Drug Authority (NDA) as a corporate body in Uganda to regulate the sale and approval of drugs on the local market, but excludes medical devices, and yet there is no similar independent body mandated to regulate medical devices.

Similar gaps are noticeable in sub-Sect. 4.1.2 of the National Drug Policy and Authority (Conduct of Clinical Trials) Regulations, 2014. It is highlighted in the guidelines that “*There is no provision under the current Act or Regulations to authorize clinical trials involving investigational medical devices.*” This is repeated in the review of the very guidelines in 2019 with minor changes introduced to include surgical appliances (e.g., tongue depressors, forceps) and sundries like medical gloves [14]. This regulatory regime is evidently not favorable for medical device translation.

Strengthening health innovation systems can be done through policies that support health research systems and a local incentive structure that focuses research on local health challenges. Other aspects of developing health innovation systems would include developing local scientific and biomedical research capacities, assessment, monitoring and standardization capabilities for medical devices in the country [15, 16].

### Establishments and ownership

Our findings indicate that majority of the respondents did not have intellectual property for their innovations. The Uganda Registration Services Bureau (URSB) is responsible for registering intellectual property in Uganda, including patents, utility model, copyrights, and trademarks, among others. There are also legal guidelines like the Uganda National Intellectual Property Policy of 2019 [17], the Trademark Act 2010 [18], and the East African Community Regional Policy for Intellectual Property [19]. All these intend to encourage technical innovation and to promote the industrial and commercial use of technical inventions and innovations, including medical devices, so as to contribute to the social, economic, industrial, and technological development of the community. However, not many medical device IPs are registered for reasons including the costs of filing the IP, the limited number of medical device innovators, and the lack of ingenuity and creativity among interested partners [20]. This limits ownership and the ability to commercialize innovations.

### Financing and support

From our study, very few medical device innovators had established companies and business models, and the majority of the innovators were based in academic institutions with weak or no links to industry partners. In this case, the go to sources of financing are grant funding, government support, and personal savings, since venture capital (VC) firms attract only well prepared innovators with registered companies, unique business models, good financials, and a convincing proposition. This explains why the majority (75%, n = 9) of the innovators had their projects financed through grants and prizes, while 25% (n = 3) used personal savings.

Additionally, there are very few VC firms investing in medical device development in Africa, leading to a limited amount of investment cash inflow into the continent. Although there is limited data, the International Finance Corporation (IFC) indicates that Africa received only $5.2 billion of the total $330 billion in venture capital in 2021, with the fintech sector taking the lion’s share [21].

The challenges in financing further stretch the Valley-of-Death (VoD) for medical device innovations originating from LMICs or targeting poverty-related diseases. The phenomenon of VoD involves a perilous period before a new business reaches a break-even point, and this is very difficult for LMIC based innovations whose business models are social impact driven, as opposed to what is preferred by traditional investors who are profit-oriented.

### Partnership

Our findings show that the majority of the innovation teams comprised only engineers. However, these innovators understood the importance of collaboration and were interested in forming partnerships with clinicians, businesspersons, industrial partners, funders, or legal practitioners. Indeed, the respondents who had identified themselves as policy makers understood their role in the translation pathway and were interested in collaborating with innovators since they had to monitor innovations to ensure they were produced to conform to standards. There was a role for social scientists in understanding community perceptions of innovation, addressing ethical concerns, and participating in post-market evaluation.

These results concur with those from a previous study by Mensah and Czajkowski, which reiterated that collaboration with social scientists, policymakers, and funders, among others, is important at various stages of translation [8, 22]. Collaborations enable continuous product development, successful navigation of regulatory pathways [23, 24], and the establishment and growth of MedTech companies. Adopting interdisciplinarity and promoting collaborative workflows to accelerate innovation will enable innovators in LMICs to reap the benefits of their creativity. Governments, academic institutions, and professional bodies should institute programs to support innovators in the MedTech translation process.

### Limitations

Despite the increase in the number of innovators in Uganda, there is only a small number concentrating on medical device innovations, and they are scattered across the country. There is no central organisation to advocate for medical device innovators, and the online community of Ugandan innovators is scattered across platforms, mostly WhatsApp. Additionally, the paucity of documentation made it difficult to identify and target key stakeholders in the field. Similarly, it was difficult to identify representatives from the industry, legal, and business sectors. Unclear regulations and policies governing medical device innovations, and their translation do not motivate the innovators’ participation in this sector.

## Conclusion

A more comprehensive study is required to determine the number of medical device innovators in Uganda and Africa at large in order to propel the translation of medical device innovation to the bedside. Support for innovators should also be provided to ensure interdisciplinary collaboration with other sectors like social scientists, industrial partners, and legal personnel for the proper translation of medical device innovations.

### Electronic supplementary material

Below is the link to the electronic supplementary material.

Supplementary Material 1

## Data Availability

The datasets used and analysed during the current study are available from the corresponding author on reasonable request.

## Abbreviations

LMIC: Low and Middle Income Countries
HIC: High Income Countries
IFC: International Finance Corporation
