# Supplementary material for: Translating medical device innovations to market - a Ugandan perspective
Source: BMC Res Notes. 2023 Oct 9;16:262. doi: 10.1186/s13104-023-06541-6 (PMC10563284; doi:10.1186/s13104-023-06541-6)
Supplement: Supplementary file 1 — Supplementary Material 1 [file 13104_2023_6541_MOESM1_ESM.docx]

**Questionnaire for Quantitative Interview:**

**Translating Medical Device Innovations to Market - a Ugandan perspective**

**Introduction:**

Dear Respondent, we are a team of researchers from Makerere University College of Health Sciences (MakCHS) working in collaboration with the University of Edinburgh conducting a study about translating medical device innovations to market in Uganda and the region. This questionnaire seeks to map out current bottlenecks in medical device innovation in Uganda, a LMIC in Sub-Saharan East Africa.

**Introductory Questions:**

1. **Which of the following categories best describes your primary profession?**
2. Innovator
3. Policy Makers/Regulators/Administration
4. Business
5. Legal
6. Social Scientist
7. Others (please specify…………………….)
8. **Have you ever participated in any medical technology innovation before?**
   1. Yes
   2. No

**Section A: Questions directed to medical device innovators.**

1. **How would you associate your innovation with?**
   1. Academia origin
   2. Industry origin
2. **At what level/stage is your most advanced innovation?**
   1. Ideation and conceptualization
   2. Proof of concept/Prototype
   3. Preclinical validation.
   4. Minimum Viable Product
   5. Clinical validation stage
   6. Pre-Market entry/Commercialization for market entry
   7. Post market
3. **How long have you been working on your innovation?**
   1. Less than one year
   2. 1-2 years
   3. 2-3 years
   4. 3-4 years
   5. 4-5 years
   6. Above 5 years
4. **What is the composition of your team working on the innovation? (select all that apply)**
   1. Clinician
   2. Engineer (biomedical, electrical, computer, etc)
   3. Social scientist
   4. Business specialist
   5. Academician
   6. Industry based partner
   7. Legal and or IP expert
   8. Others….. specify
5. **Which partnership are you interested in currently given the stage of your innovation?**
   1. Legal
   2. Engineering
   3. Social science
   4. Academician
   5. Industrial partner
   6. Business person
   7. Clinician and healthcare based
   8. Others, specify
6. **Have you generated any Intellectual Property (IP) from your innovation?**
   1. Yes
   2. No
7. **If yes, what IP do you have?**
   1. Patent
   2. Copy right
   3. Utility Model
   4. Trade mark
   5. Design rights
   6. Others, Specify
8. **Have you formed a company out of your innovation?**
   1. Yes
   2. No
9. **What is your major source of funding towards your innovation**
   1. Grants and prizes
   2. Personal savings
   3. Joint group investments
   4. Philanthropists
   5. Through revenue (sales, IP, etc)
   6. Others
10. **Have you already developed a business model for your innovation?**
    1. Yes
    2. No
11. **How do you plan to commercialize your innovation?**
    1. Through forming a spin out company
    2. Licensing IP to other interested parties
    3. Franchising the innovation
    4. Obtaining a commercial loan
12. **Do you have any other comments for innovators in the medical field who are seeking to have their innovations translated to market?** ………………………………………………………………………………………………………………………………………………………………………………………………….

**Section B: Regulations around medical devices translation**

- - - 1. **What do you think is your role in the translational pathway of innovations?** (select all that apply)

1. Development of Med-Tech Translation Pathway guidelines or standards.
2. Ensuring that guidelines are followed by the innovators.
3. Monitoring innovations to ensure safe and effective medical devices are produced to conform to standards.
4. Validation and verification of the innovations
5. Protecting the rights of the innovators
6. Providing resources to innovators like funds, technical expertise, etc
7. Other (please specify…………………………)
8. **Are there documented policies governing the translation of innovations into marketable products?**
9. Yes
10. No
11. Not sure

**If yes, give some examples of those documented policies governing the translation of innovations into marketable products?**

……………………………………………………………………………………….

**If yes, where can the innovators find these policies?**

1. Websites, specify.
2. Physically at the offices
3. Library
4. Other (Please specify)

**If no, can you give examples of policies that you think should be put in place?**

……………………………………………………………………………………………………………

1. **Which partners does your entity have in the Med-Tech Translational Pathway?** (Select all that apply)
2. National Drug Authority
3. Uganda National Bureau of Standards (UNBS)
4. Ministry of Health
5. Uganda National Council for Science and Technology (UNCST)
6. Innovation hubs
7. Professionals’ Association societies (Like Pharmaceutical society, UNAHME- Uganda National Assoxiation of Hospital and Medical Engineers, etc)
8. Some international bodies like ISO, IEEE (Institute of Electrical and Electronic Engineers)
9. Academic partner
10. Clinical partner
11. Other (Please specify……………………. )
12. **What are the necessary requirements for Med-Tech Translation to Market?** (Select all that apply)
13. Ethical Approval from a Local Institutional Review Board (IRB)
14. Approval from Uganda National Council for Science and Technology (UNCST)
15. Approved Clinical Trials’ process
16. Approval from National Drug Authority (NDA)
17. Approval from Uganda National Bureau of Standards (UNBS)
18. Approval from Uganda Registration Service Board (URSB)
19. I don’t know
20. Other (Please specify)
21. **Are there guidelines or regulations for premarket and post market approval of medi-tech devices in the country?**
22. Yes
23. No
24. Not relevant
25. **Does your organisation support innovators in the Med-Tech Translation process?**
26. Yes
27. No
28. Not applicable
29. **If yes, in what way?**
30. Availing innovators with guidelines to follow during the entire process.
31. Providing innovators with collaboration avenues with different cadres in industry.
32. Intellectual property protection.
33. Incubation of the innovations
34. Providing funds
35. Other
36. **Why do you think some innovations fail to translate into marketable products? (select most three)**
37. Limited knowledge of the Med-Tech Translation process.
38. Limited funding
39. Delayed approvals
40. Targeting the wrong market
41. Limited human resource and diversity
42. Limited government support
43. Limited manufacturing capacity
44. Other
45. **Would you be interested in future collaborations in line with the Med-tech Translation pathway?**
46. Yes
47. No
48. **Do you have any other comments for innovators in the medical field who are seeking to have their innovations translated to market?** ………………………………………………………………………………………………………………………………………………………………………………………………….

**Section C: Questions directed to a Social Scientist (**To be answered by those that have never participated in a medical devices innovation before and those who are under the social science category and the other speciality category**)**

- - - 1. **Do you think it is important to have a Social Science expert to take part in the regulation of medical devices?**

1. Yes
2. No

If yes why?............................

- - - 1. **What do you think is your role in the translational pathway of innovations? (select all that apply)**

1. Evaluation of innovations in Med-Tech conform to the standards.
2. Regulation of innovations in Med-tech
3. Evaluation of post-market feedback about Med-Tech innovations.
4. Understanding community perception about the innovation.
5. Educating the community about the innovation and the regulations involved.
6. Recall innovations in Med-Tech on market
7. Other (please specify) …………………………..
   - - 1. **How best do you think you can help innovators during the Med-Tech translation process? (Select all that apply)**
8. Providing collaboration avenues and linking them up with manufacturers in industry.
9. Provide guidelines to follow to conform to standards.
10. Provide mentorship to the innovators.
11. Provide guiding information regarding the on post-market expectations.
12. Other (please specify) ………………………….
    - - 1. **Which of the following will be of major concern to you in translation of medical technologies? (choose most 3)**
      1. How end users will interact with the device.
      2. Does it look good?
      3. How the device addressed the challenge presented.
      4. How safe is the device.
      5. Has the device obtained the necessary approvals.
      6. What’s new with your device that other devices don’t have.
      7. Is it affordable?
      8. Does it address gender concerned issues?
         1. **Would you be interested in future collaborations?**
         2. Yes
         3. No
         4. **Do you have any other comments for innovators in the medical field who are seeking to have their innovations translated to market?** ………………………………………………………………………………………………………………………………………………………………………………………………….

**Thank you for your time.**
